# Rapid Detection of Piperacillin-Tazobactam Resistance in Klebsiella pneumoniae and Escherichia coli

**DOI:** 10.1128/spectrum.04366-22

**Published:** 2023-02-14

**Authors:** José Manuel Ortiz de la Rosa, Ángel Rodríguez-Villodres, María Adelina Gimeno Gascón, Guillermo Martín-Gutiérrez, José Miguel Cisneros, José Antonio Lepe

**Affiliations:** a Clinical Unit of Infectious Diseases, Microbiology and Parasitology, University Hospital Virgen del Rocío, Seville, Spain; b Institute of Biomedicine of Seville (IBiS), University Hospital Virgen del Rocío/CSIC/University of Seville, Seville, Spain; c Centro de Investigación Biomédica en Red de Enfermedades Infecciosas (CIBERINFEC), Madrid, Spain; d Department of Medicine, University of Seville, Seville, Spain; e Department of Microbiology, University of Seville, Seville, Spain; The University of North Carolina at Chapel Hill

**Keywords:** piperacillin-tazobactam, *Klebsiella pneumoniae*, *Escherichia coli*, RapidTZP test, empirical therapy

## Abstract

Rapid determination of susceptibility to piperacillin-tazobactam (TZP) is very important since the development of antibiotic resistance and inadequate treatment could increase the risk of clinical failure in infected patients, especially if such resistance is unknown to the clinician. Therefore, based on color change from orange to yellow of phenol red due to glucose metabolism (bacterial growth) in the presence of an adequate concentration of TZP (10 mg/L piperacillin and 5 mg/L tazobactam), the RapidTZP test has been developed to detect TZP resistance in Escherichia coli and Klebsiella pneumoniae isolates in a maximum of 3 h. A total of 140 isolates, 43 of E. coli and 97 of K. pneumoniae, were used to evaluate the performance of the test, 60 being resistant to TZP. The sensitivity and specificity of the test were 98.24% and 100%, respectively. Additionally, the RapidTZP test was validated by a pellet obtained directly from blood culture bottles. A total of 37 positive blood cultures for E. coli and 43 for K. pneumoniae were used for validation, 8 of them resistant to TZP. The sensitivity and specificity shown in the evaluation were 100% for both parameters. This new test is easy, fast, and accurate, providing results in 3 h.

**IMPORTANCE** TZP is an antibiotic widely used for the empirical treatment of severe infections such as bloodstream infections. However, resistance to TZP in K. pneumoniae and E. coli has been increasing in the last few years. Thus, rapid detection of TZP resistance is critical to optimize the empirical treatment of patients with severe infections. In this study, we developed and evaluated a rapid test (RapidTZP) for the detection of TZP resistance in K. pneumoniae and E. coli directly from positive hemocultures in just 3 h. This rapid test has been validated on 138 K. pneumoniae and E. coli clinical isolates directly from agar plates and 80 K. pneumoniae and E. coli isolates causing bloodstream infections. The results demonstrate that the RapidTZP test has great clinical potential to optimize the empirical treatment of patients with bloodstream infections.

## INTRODUCTION

Antimicrobial resistance is an emerging global threat that requires urgent measures (1). Piperacillin-tazobactam (TZP) is a broad-spectrum β-lactam–β-lactamase inhibitor (BL-BLI) combination frequently used to treat severe infections and health care-associated infections (2, 3). TZP is considered by some authors as a reliable option for the treatment of severe infections by extended-spectrum-β-lactamase (ESBL)-producing Gram-negative bacilli (ESBL-GNB), to reduce the use of carbapenems, which could facilitate the appearance and spread of carbapenemases (4, 5). However, abusive use of TZP has led to the appearance of resistant strains (6, 7). Recent studies have reported that TZP-resistant Escherichia coli is becoming increasingly prevalent. Data extracted from the Study for Monitoring Antimicrobial Resistance Trends (SMART) from 2002 to 2010 and 2016 to 2017 revealed an increase in the rate of TZP resistance in intra-abdominal E. coli isolates, from 7.7% to 10.0% (8, 9). These data are more worrying in Klebsiella pneumoniae, where TZP resistance increased from 11.7% in 2002 to 2010 to 33.4% in 2016 to 2017 (8, 9).

Inadequate antimicrobial treatment led to a longer hospital stay and a higher rate of mortality (10–14). Currently, the standard reference technique for determining susceptibility to TZP in many clinical laboratories is based on broth microdilution through semiautomated methods, which require 18 to 20 h to obtain results (i.e., MicroScan WalkAway and Vitek). Other methods, such as disk diffusion and MIC gradient strips, can also be used but also require at least 18 h (15). In this sense, developing a rapid test for the detection of TZP resistance is crucial to optimize the use of this antibiotic, with the consequent improvement of the patient’s outcome. In this study, we have developed a rapid (3-h) and cost-effective test, the RapidTZP test for K. pneumoniae and E. coli, which might be used worldwide, regardless of the technical level of the laboratory.

## RESULTS

For the development step, a total of 138 isolates were tested to evaluate the performance of the RapidTZP test. Among the 60 TZP-resistant isolates, 30 were ESBL producers (29 *bla*_CTX-M_ and 1 *bla*_SHV_+*bla*_TEM_), 27 exhibited AmpC (4 *bla*_CMY_, 5 *bla*_DHA_, and 18 *bla*_AmpC_) as an acquired mechanism of resistance, and 3 were positive for both ESBL and AmpC (1 *bla*_CTX-M_+*bla*_CMY_ and 2 *bla*_CTX-M_+*bla*_AmpC_) through phenotypic and/or genotypic characterization (see Table S1 in the supplemental material). Samples presented above harboring *bla*_AmpC_ were determined by phenotypic characterization but remained negative for all *ampC* genes tested (*bla*_CMY_ and *bla*_DHA_). On the other hand, the 78 TZP-susceptible isolates were grouped into 28 wild-type isolates, 27 ESBL-producing isolates (25 *bla*_CTX-M_, 1 *bla*_SHV_, and 1 *bla*_SHV_+*bla*_TEM_), 9 TEM-producing E. coli isolates, and 16 AmpC-producing isolates (11 *bla*_DHA_ and 5 *bla*_AmpC_). Other mechanisms of resistance were not further investigated. Among the TZP-susceptible isolates (MICs of TZP, 0.06 to 8 mg/L), 78 gave negative results with the RapidTZP test (Table S1). All the TZP-resistant isolates regardless of their mechanism of resistance to TZP (*n* = 60; MICs of TZP, 12 to >128 mg/L) had a positive test result, except for a single isolate of K. pneumoniae (isolate C3.29 showing a MIC of TZP of >128 mg/L) which gave a negative result. However, this isolate showed colonies inside the TZP gradient strip inhibition zone, suggesting the presence of a minor subpopulation resistant and one major subpopulation susceptible (major error) (Table 1 and Table S1). Thus, the development step showed a strong correlation between the RapidTZP test and the standard method (MicroScan system WalkAway), for both susceptible and resistant isolates (Table 1). The sensitivity, specificity, positive predictive value (PPV), and negative predictive value (NPV) were found to be 99.12%, 100%, 100%, and 98.78%, respectively (Table 1).

**TABLE 1:** Development of the RapidTZP test in the detection of K. pneumoniae and E. coli TZP-resistant isolates^*a*^

Microorganism	No. of isolates with TZP MIC (mg/L) of:		No. of isolates negative or positive for resistance mechanism								No. of isolates with rapid test result:		% sensitivity (95% CI)	% specificity (95% CI)	% PPV	% NPV
			Resistant isolates				Susceptible isolates									
	≤8	>8	−	ESBL	AmpC	ESBL + AmpC	−	ESBL	AmpC	ESBL + AmpC	POS	NEG				
K. pneumoniae	41	56	0	26	27	3	1	24	16	0	55	42	98.24 (90.6–99.7)	100 (91.4–100)	100	97.56
E. coli	39	4	0	4	0	0	36	3	0	0	4	39	100 (51–100)	100 (91–100)	100	100

a −, negative; POS, positive; NEG, negative; CI, confidence interval.

The evaluation step was performed with 80 clinical samples recovered from blood cultures of patients with bacteriemia, to confirm the effective performance of the RapidTZP test in the routine clinical practice of the microbiology department. Eight out of the 18 samples were resistant (MICs of TZP, 24 to >256 mg/L), giving positive results with the RapidTZP test (Table 2). The group of 8 resistant isolates encompassed 7 of K. pneumoniae (5 non-ESBL, 1 ESBL [1 *bla*_CTX-M_], and 1 carbapenemase [*bla*_OXA-48_]) and 1 of E. coli (1 ESBL [1 *bla*_CTX-M_]) (Table S2). On the other hand, in the 72 TZP-susceptible isolates (54 non-ESBL and 18 ESBL), the presence of the *bla*_CTX-M_ gene was responsible for all ESBL phenotypes (Table S2). Statistical analysis of the results showed an excellent accuracy of the RapidTZP test, raising sensitivity, specificity, and predictive values to 100% (Table 2).

**TABLE 2:** Evaluation of the RapidTZP test from positive blood cultures in the detection of TZP-resistant isolates^*a*^

Microorganism	No. of isolates with TZP MIC (mg/L) of:		No. of isolates negative or positive for resistance mechanism				No. of isolates with rapid test result:		% sensitivity (95% CI)	% specificity (95% CI)	% PPV	% NPV
			Resistant isolates		Susceptible isolates							
	≤8	>8	−	ESBL	−	ESBL	POS	NEG				
K. pneumoniae	36	7	5	2	19	17	7	36	100 (64.6–100)	100 (90.4–100)	100	100
E. coli	36	1	0	1	35	1	1	36	100 (20.7–100)	100 (90.4–100)	100	100

a Symbol and abbreviations: −, negative; POS, positive; NEG, negative; CI, confidence interval.

## DISCUSSION

In this study, we developed a rapid colorimetric assay, named the RapidTZP test, for detecting TZP resistance in K. pneumoniae or E. coli regardless of the mechanism of resistance. A collection of 138 clinical isolates of K. pneumoniae and E. coli were used to evaluate the test. Moreover, a clinical evaluation was performed using 80 samples recovered from blood cultures in routine clinical practice. Discordant results in TZP MICs between MicroScan and standard MIC gradient strips were observed for five (2.27%) of the isolates studied. Those discordant values were found to be surrounding the breakpoint value, being corrected after a second replicate of the standard MIC gradient strips. Probably those discordant results were obtained due to a higher inoculum used for the MIC gradient strip performance (16).

This study showed that the detection of some resistance determinants through molecular techniques, such as quantitative PCR (qPCR) or hybridization, is not necessarily leading to a resistance phenotype for certain antibiotics like TZP, which is widely used in clinical practice as empirical treatment. Except the *bla*_CMY_ gene, which was strongly associated with resistant isolates, the other resistance determinants were detected in both sensitive and resistant isolates. Similar data were observed in another study, in which TEM or ESBL production was not always associated with TZP resistance in E. coli or K. pneumoniae, respectively (17, 18).

Of note, the RapidTZP test is very sensitive and specific and has high PPV and NPV, being able to detect all the TZP-resistant K. pneumoniae and E. coli isolates analyzed in this study. Only one resistant K. pneumoniae isolate was not detected by the RapidTZP test. MIC gradient strip results showed inner colonies in the inhibition zone, which were responsible for the high MIC value. Thus, one possible explanation is that there were two populations in this sample, one susceptible (the major) and one resistant (the minor) and the test was not able to detect this in 3 h, because the concentration of the resistant population was too low. More studies are needed to confirm this hypothesis.

Other similar tests have been developed for the detection of TZP resistance, such as the extended-spectrum resistance to beta-lactam/beta-lactamase inhibitors (ESRI) test (19), which was developed for detecting TZP-resistant isolates but also TZP-susceptible isolates able to develop TZP resistance (ESRI developers), which are primarily susceptible to TZP (19). The main difference from these interesting tests is that the RapidTZP test can detect resistance independently of the resistance mechanism used by the bacteria. In contrast, the ESRI test is based on the detection of β-lactam hydrolysis through the production of a certain β-lactamase enzyme; consequently, other resistance mechanisms could not be detected by the ESRI test. Moreover, the ESRI test has more experimental steps, requires higher concentrations of β-lactam antibiotics (300×), and is exclusive to E. coli. Thus, RapidTZP is simpler, less expensive, and more sensitive than the ESRI method. In addition, manual and automatic methods like rapid antimicrobial susceptibility testing (RAST) and the automated commercial laser-scattering-based *in vitro* system Alfred 60AST have been developed to identify TZP-resistant E. coli from positive blood culture bottles within 4 to 6 h (20, 21). Meanwhile, other tests with similar approaches have been developed, such as the Rapid Polymyxin NP, which was commercialized and was based on the resazurin color change in response to the bacterial growth in the presence of a polymyxin drug, or the rapid fosfomycin/Escherichia coli NP test, which was developed to detect fosfomycin resistance in E. coli isolates through an orange-to-yellow color change of red phenol (22, 23). Unlike the latter methods, RapidTZP uses 10 μL from an inoculum with a 0.5 McFarland standard, being lower than the 3 to 3.5 McFarland standard used by the Rapid Polymyxin NP and the rapid fosfomycin/Escherichia coli NP test. This reduced inoculum could shorten the time to obtain a result, because clinical samples with less bacterial inoculum can be tested.

**FIG 1:**
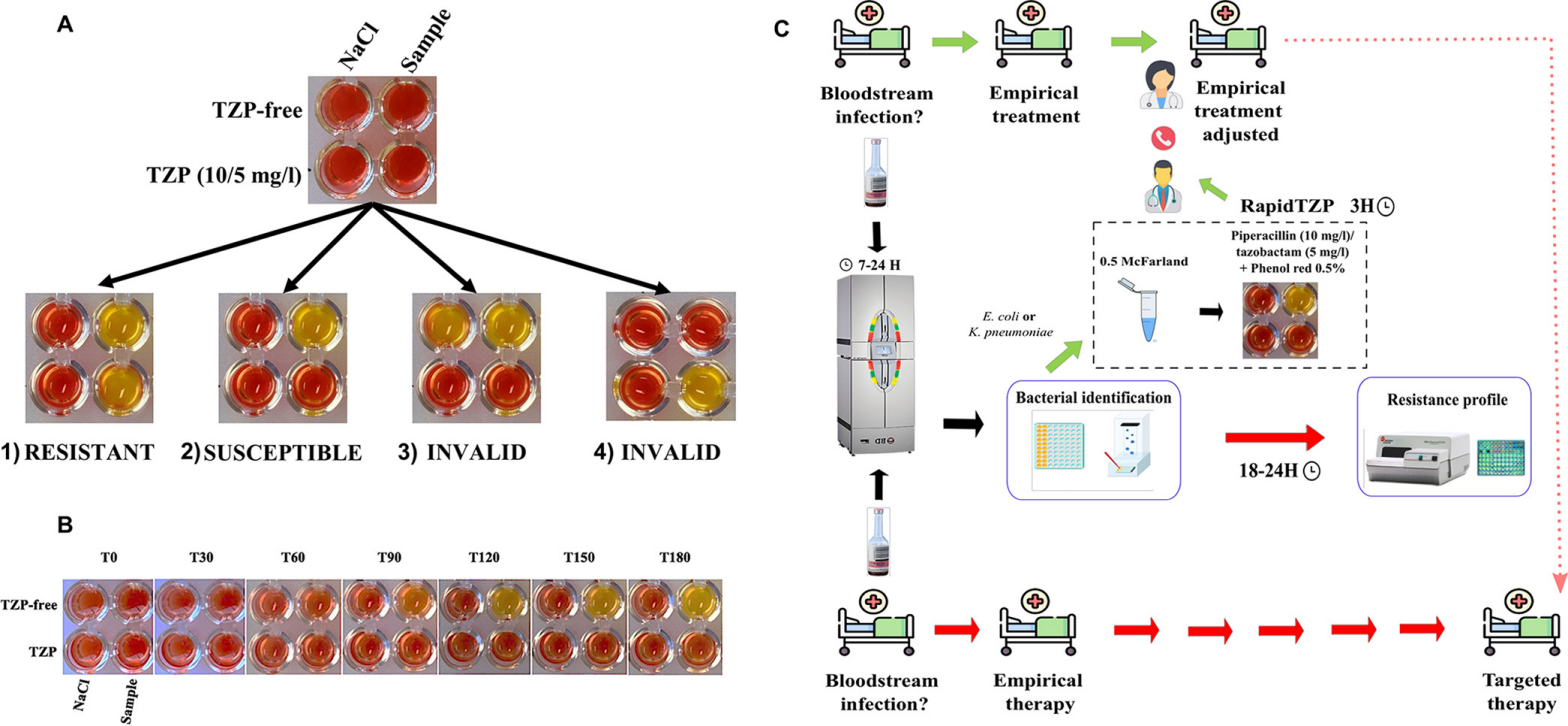
(A) RapidTZP test, representative results. The first column shows noninoculated wells, containing the test solution with and without TZP and NaCl. In column 2, the solution test with and without TZP was inoculated with the sample. After 3 h of incubation at 37°C, four possible results are represented in the lower part of the figure. (1) Second column yellow, isolate grew in the presence and the absence of TZP. Thus, it was TZP resistant. (2) As negative control, isolate grew (yellow color) in the absence of TZP and did not grow (orange color) in the presence of this antibiotic. Thus, it was TZP susceptible. (3 and 4) An invalid test result could be found when the isolate grew in the first column (contamination of the medium) or the isolate grew (yellow color) in the presence of TZP and did not grow (orange color) in the absence of this antibiotic (inoculation mistake or contamination). (B) Change of color versus time every 30 min for a susceptible isolate (second column) and noninoculated wells (first column). (C) Flowchart of the RapidTZP methodology and its possible application in clinical practice.

The introduction of the RapidTZP test into clinical practice would have clinical implications, being essential to establish an adequate antibiotic treatment especially during the first 24 h, when the use of TZP is especially relevant (Fig. 1C) (12–14). This might avoid a possible therapeutic failure due to the development of resistant bacteria, as well as an inappropriate use of antibiotics that in turn could contribute to increasing the rates of resistance of BL-BLI. Hence, a further clinical prospective study will be performed through the implementation of the RapidTZP test in routine clinical practice in order to assess the impact of the RapidTZP test on the clinical outcomes of patients with bloodstream infection caused by K. pneumoniae or E. coli. To this end, we need to determine mainly the time until the change of antibiotic treatment, and other factors such as the duration of symptoms, clinical cure, complications of infection, length of hospital stay, and mortality.

The RapidTZP test was designed for K. pneumoniae and E. coli isolates growing in blood culture bottles or from agar plates. Although those bacterial species are the main Gram-negative bacteria isolated from blood, further experiments are necessary to evaluate or adapt the test to other bacterial genera and different clinical samples. Besides, the RapidTZP test is interpreted through the detection of a color change with the naked eye. Therefore, despite the fact that resistant isolates clearly change from orange to yellow, low-level resistance isolates may require a more precise reading and/or a second reader.

In conclusion, the RapidTZP test is a rapid and easy-to-perform test, combining excellent sensitivity and specificity. The use of this system is particularly interesting in a context of increased prevalence of ESBL producers, among which resistance to TZP seems to be higher year by year, reaching resistance rates of 30%, as reported in a previous study (8, 9). It could provide a novel contribution to microbiological diagnostics, enabling us to determine TZP resistance in less time and provide improved clinical prognosis, in terms of both survival and the lack of recurrence of resistant bacteria, as well as produce a positive economic impact on the health care system.

## MATERIALS AND METHODS

### Bacterial strains.

A total of 140 isolates, encompassing K. pneumoniae and E. coli, collected from clinical samples from the Microbiology Service at the University Hospital Virgen del Rocío (Seville, Spain) were used in the development of the test. Identification of the isolates was performed using matrix-assisted laser desorption ionization–time of flight (MALDI-TOF) (Bruker, Germany). This collection included 60 TZP-resistant isolates and another 80 TZP-susceptible isolates (Table 1). Additionally, the test was clinically validated on 80 blood cultures positive for K. pneumoniae (*n* = 43) and E. coli (*n* = 37) clinical isolates recovered over a 3-month period. K. pneumoniae ATCC 10031 and E. coli ATCC 25922 were used as negative controls. Four TZP-resistant isolates from the evaluation step (2 of E. coli and 2 of K. pneumoniae) were included as positive controls in the clinical validation.

### Antimicrobial susceptibility testing.

MIC values of TZP were determined by two methods: (i) the semiautomatic system MicroScan WalkAway (Beckman Coulter, USA), which is the routine method used in many hospitals for the determination of the susceptibility profile, and (ii) TZP gradient strips (Liofilchem, Italy) following the manufacturer’s instructions. All experiments were repeated in three separate experiments, using daily freshly prepared plates and inocula. Clinical breakpoints were established according to the European Committee on Antimicrobial Susceptibility Testing (EUCAST) (24). Hence, isolates with TZP MICs of ≤8 mg/L were categorized as susceptible, while those with MICs of >8 mg/L were categorized as resistant.

### Detection of β-lactamase genes.

The *bla*_CTX-M_, *bla*_TEM_, *bla*_OXA-1_, *bla*_SHV_ (E. coli), *bla*_DHA_, and *bla*_CMY_ genes were analyzed in the isolates used in the evaluation step of the RapidTZP test by singleplex qPCR using the primers and cycling conditions listed in Table S3 in the supplemental material.

### Reagents and solutions.

The rapid test requires five reagents, namely, Mueller-Hinton broth (MHB), d-(+)-glucose monohydrate, 0.5% phenol red solution, piperacillin sodium salt (powder), and tazobactam sodium (powder) (Sigma-Aldrich, UK). The test solution was prepared by mixing 47 mL of MHB, 700 μL of 0.5% phenol red solution, and 2.5 mL of d-(+)-glucose (10%). The pH was further adjusted to 7.5, and the solution was autoclaved to obtain a final concentration of 2.5% MHB-CA (cation-adjusted) powder, 0.007% phenol red solution, and 0.5% d-(+)-glucose. This solution can be kept at 4°C for 1 week or at −80°C for 6 months and must be prewarmed at 37°C before use to prevent growth delay and a delayed color change. Before performing the experiment, piperacillin and tazobactam were added to the test solution and mixed to obtain a final piperacillin-tazobactam concentration of 10/5 mg/L.

### Bacterial preparation.

In the development step, bacterial colonies were resuspended into 2 mL of sterile NaCl (0.85%) to obtain a 0.5 to 1 McFarland standard optical density (10^8^ CFU/mL) using a turbidimeter. In the evaluation step, bacterial pellets obtained from positive blood cultures were used as in the development step to obtain a 0.5 to 1 McFarland standard optical density (10^8^ CFU/mL). A bacterial suspension was prepared for each isolate to be tested.

### Test and reading.

Inoculation and reading of the results were performed as previously described by Nordmann et al. for colistin and fosfomycin with slight changes (22, 23). Briefly, bacterial suspension was inoculated into a round-base 96-well polystyrene micro-test plate (reference 82.1582.001; Sarstedt, Nümbrecht, Germany) in the presence or absence of antibiotics, in separate wells. A total of 10 μL of bacterial suspension was added to both wells, corresponding to a final bacterial concentration of 10^6^ CFU/mL in each well. The first well contained 150 μL of test solution without TZP, and the second contained 150 μL of test solution supplemented with 10 mg/L piperacillin and 5 mg/L tazobactam. Another couple of wells were prepared with the same combination and inoculated with 10 μL of NaCl to have a medium control (Fig. 1). The inoculated plate was incubated at 37°C without shaking. The color change was checked every 30 min, and the time to read the results was established at 3 h, based on the maximum time needed to obtain a stable result for all tested isolates (Fig. 1A and B). The result was considered positive if the isolate grew in the presence of TZP, and if a color change from red-orange to yellow was observed in the wells, confirming the metabolism of glucose by the isolate (Fig. 1). Conversely, a test result was considered negative when the isolate did not grow in the presence of TZP, meaning the color of the wells remained red-orange.
