# Supplementary material for: Rapid Detection of Piperacillin-Tazobactam Resistance in Klebsiella pneumoniae and Escherichia coli
Source: Microbiol Spectr. 2023 Feb 14;11(2):e04366-22. doi: 10.1128/spectrum.04366-22 (PMC10100654; doi:10.1128/spectrum.04366-22)
Supplement: Supplemental file 1 — Supplemental material. Download spectrum.04366-22-s0001.pdf, PDF file, 0.3 MB [file spectrum.04366-22-s0001.pdf]

**Table S1**. Individualized results of the RapidTZP test for 138 *K. pneumoniae* and *E. coli* clinical isolates.

| Name | Microorganism | MIC TZP (µg/ml) | Resistance phenotype | Resistance determinant |
|---|---|---|---|---|
| C1.9 | *Klebsiella pneumoniae* | 32 | ESBL | CTX-M TEM |
| C1.1 | *Klebsiella pneumoniae* | 8 | ESBL | CTX-M |
| C1.5 | *Klebsiella pneumoniae* | 3 | ESBL | CTX-M TEM |
| C1.7 | *Klebsiella pneumoniae* | 3 | ESBL | CTX-M |
| C1.25 | *Klebsiella pneumoniae* | 1 | AmpC | DHA |
| C1.33 | *Klebsiella pneumoniae* | >128 | AmpC | Other AmpC |
| C1.34 | *Klebsiella pneumoniae* | >128 | AmpC | Other AmpC |
| C1.35 | *Klebsiella pneumoniae* | 2 | AmpC | DHA |
| C1.80 | *Klebsiella pneumoniae* | 2 | AmpC | Other AmpC |
| C2.25 | *Klebsiella pneumoniae* | >128 | AmpC | Other AmpC |
| C3.4 | *Klebsiella pneumoniae* | 16 | AmpC | Other AmpC |
| C3.28 | *Klebsiella pneumoniae* | >128 | ESBL | CTX-M |
| C3.29 | *Klebsiella pneumoniae* | >128 | ESBL | CTX-M |
| C3.36 | *Klebsiella pneumoniae* | >128 | ESBL | CTX-M |
| C3.54 | *Klebsiella pneumoniae* | 4 | AmpC | DHA |
| C4.4 | *Klebsiella pneumoniae* | 32 | AmpC | DHA |
| C4.12 | *Klebsiella pneumoniae* | 4 | AmpC | DHA |
| C4.15 | *Klebsiella pneumoniae* | 3 | AmpC | DHA |
| C4.43 | *Klebsiella pneumoniae* | >128 | AmpC | Other AmpC |
| C4.58 | *Klebsiella pneumoniae* | 1 | AmpC | DHA |
| C4.59 | *Klebsiella pneumoniae* | >128 | AmpC | Other AmpC |
| C4.76 | *Klebsiella pneumoniae* | 4 | AmpC | DHA |
| C4.77 | *Klebsiella pneumoniae* | >128 | AmpC | Other AmpC |
| C5.20 | *Klebsiella pneumoniae* | 4 | AmpC | DHA |
| C5.37 | *Klebsiella pneumoniae* | >128 | ESBL + AmpC | CTX-M + CMY |
| C5.38 | *Klebsiella pneumoniae* | >128 | ESBL + AmpC | CTX-M + Other AmpC |
| C5.39 | *Klebsiella pneumoniae* | >128 | ESBL + AmpC | CTX-M + Other AmpC |
| C5.45 | *Klebsiella pneumoniae* | >128 | AmpC | CMY |
| C5.46 | *Klebsiella pneumoniae* | 64 | AmpC | DHA |
| C5.62 | *Klebsiella pneumoniae* | >128 | AmpC | Other AmpC |
| C5.74 | *Klebsiella pneumoniae* | 64 | AmpC | DHA |
| C5.75 | *Klebsiella pneumoniae* | 128 | AmpC | DHA |
| C6.1 | *Klebsiella pneumoniae* | 12 | AmpC | DHA |
| C6.22 | *Klebsiella pneumoniae* | 128 | ESBL | CTX-M TEM |
| C6.32 | *Klebsiella pneumoniae* | 0.06 | ESBL | SHV TEM |
| C6.39 | *Klebsiella pneumoniae* | >128 | AmpC | Other AmpC |
| C6.53 | *Klebsiella pneumoniae* | >128 | AmpC | CMY |
| C6.57 | *Klebsiella pneumoniae* | >128 | AmpC | TEM |
| C6.58 | *Klebsiella pneumoniae* | >128 | AmpC | Other AmpC |
| C6.59 | *Klebsiella pneumoniae* | 4 | AmpC | Other AmpC |
| C6.60 | *Klebsiella pneumoniae* | >128 | AmpC | CMY |
| C7.2 | *Klebsiella pneumoniae* | >128 | AmpC | CMY |

| | | | | |
|---|---|---|---|---|
| C7.7 | *Klebsiella pneumoniae* | 2 | AmpC | Other AmpC |
| C7.9 | *Klebsiella pneumoniae* | >128 | AmpC | Other AmpC |
| C7.10 | *Klebsiella pneumoniae* | >128 | AmpC | Other AmpC |
| C7.12 | *Klebsiella pneumoniae* | 3 | AmpC | DHA |
| C7.19 | *Klebsiella pneumoniae* | 2 | AmpC | Other AmpC |
| C7.31 | *Klebsiella pneumoniae* | 32 | AmpC | Other AmpC |
| C7.71 | *Klebsiella pneumoniae* | 4 | AmpC | Other AmpC |
| C8.3 | *Klebsiella pneumoniae* | >128 | AmpC | Other AmpC |
| C8.21 | *Klebsiella pneumoniae* | 3 | AmpC | DHA |
| C8.42 | *Klebsiella pneumoniae* | >128 | AmpC | Other AmpC |
| C8.46 | *Klebsiella pneumoniae* | >128 | AmpC | Other AmpC |
| C8.51 | *Klebsiella pneumoniae* | >128 | AmpC | Other AmpC |
| C8.70 | *Klebsiella pneumoniae* | 2 | ESBL | CTX-M TEM |
| C8.77 | *Klebsiella pneumoniae* | 4 | AmpC | DHA |
| C1.20 | *Klebsiella pneumoniae* | 64 | ESBL | CTX-M TEM |
| C1.21 | *Klebsiella pneumoniae* | 64 | ESBL | CTX-M TEM |
| C1.22 | *Klebsiella pneumoniae* | 128 | ESBL | CTX-M TEM |
| C1.23 | *Klebsiella pneumoniae* | 128 | ESBL | CTX-M TEM |
| C1.24 | *Klebsiella pneumoniae* | 12 | ESBL | CTX-M TEM |
| C1.27 | *Klebsiella pneumoniae* | >128 | ESBL | CTX-M TEM |
| C1.29 | *Klebsiella pneumoniae* | 16 | ESBL | CTX-M TEM |
| C1.32 | *Klebsiella pneumoniae* | 64 | ESBL | CTX-M |
| C1.40 | *Klebsiella pneumoniae* | 8 | ESBL | CTX-M TEM |
| C1.43 | *Klebsiella pneumoniae* | >128 | ESBL | CTX-M TEM |
| C1.46 | *Klebsiella pneumoniae* | 4 | ESBL | CTX-M |
| C1.52 | *Klebsiella pneumoniae* | 4 | ESBL | CTX-M |
| C1.55 | *Klebsiella pneumoniae* | 32 | ESBL | CTX-M |
| C1.56 | *Klebsiella pneumoniae* | 4 | ESBL | CTX-M |
| C1.60 | *Klebsiella pneumoniae* | 16 | ESBL | SHV TEM |
| C1.63 | *Klebsiella pneumoniae* | 2 | ESBL | CTX-M |
| C1.64 | *Klebsiella pneumoniae* | 6 | ESBL | CTX-M |
| C1.68 | *Klebsiella pneumoniae* | 24 | ESBL | CTX-M TEM |
| C1.70 | *Klebsiella pneumoniae* | 12 | ESBL | CTX-M TEM |
| C1.73 | *Klebsiella pneumoniae* | 16 | ESBL | CTX-M TEM |
| C1.79 | *Klebsiella pneumoniae* | 12 | ESBL | CTX-M TEM |
| C2.4 | *Klebsiella pneumoniae* | 1 | ESBL | SHV |
| C2.10 | *Klebsiella pneumoniae* | 24 | ESBL | CTX-M TEM |
| C2.14 | *Klebsiella pneumoniae* | 2 | ESBL | CTX-M |
| C2.16 | *Klebsiella pneumoniae* | 12 | ESBL | CTX-M TEM |
| C2.19 | *Klebsiella pneumoniae* | 2 | ESBL | CTX-M |
| C2.20 | *Klebsiella pneumoniae* | 4 | ESBL | CTX-M TEM |
| C2.21 | *Klebsiella pneumoniae* | >128 | ESBL | CTX-M TEM |
| C2.23 | *Klebsiella pneumoniae* | 32 | ESBL | CTX-M TEM |
| C2.24 | *Klebsiella pneumoniae* | 3 | ESBL | CTX-M |
| C2.28 | *Klebsiella pneumoniae* | 2 | ESBL | CTX-M |
| C2.30 | *Klebsiella pneumoniae* | 8 | ESBL | CTX-M TEM |

| | | | | |
|---|---|---|---|---|
| C2.31 | *Klebsiella pneumoniae* | 6 | ESBL | CTX-M TEM |
| C2.33 | *Klebsiella pneumoniae* | 8 | ESBL | CTX-M TEM |
| C2.35 | *Klebsiella pneumoniae* | 128 | ESBL | CTX-M TEM |
| C2.36 | *Klebsiella pneumoniae* | 24 | ESBL | CTX-M TEM |
| C2.37 | *Klebsiella pneumoniae* | 2 | ESBL | CTX-M |
| C2.40 | *Klebsiella pneumoniae* | 8 | ESBL | CTX-M TEM |
| C2.41 | *Klebsiella pneumoniae* | 0.5 | ESBL | CTX-M TEM |
| C2.42 | *Klebsiella pneumoniae* | 1 | ESBL | CTX-M TEM |
| ES-1 | *Escherichia coli* | 1.5 | - | - |
| ES-3 | *Escherichia coli* | 1 | ESBL | CTX-M |
| ES-4 | *Escherichia coli* | 24 | ESBL | CTX-M |
| ES-5 | *Escherichia coli* | 32 | ESBL | CTX-M TEM |
| ES-6 | *Escherichia coli* | 1.5 | - | TEM |
| ES-8 | *Escherichia coli* | 1 | - | - |
| ES-9 | *Escherichia coli* | 1 | - | - |
| ES-10 | *Escherichia coli* | 1 | - | TEM |
| ES-11 | *Escherichia coli* | 4 | - | - |
| ES-12 | *Escherichia coli* | 0.38 | - | TEM |
| ES-13 | *Escherichia coli* | 0.75 | - | - |
| ES-14 | *Escherichia coli* | 0.5 | - | TEM |
| ES-15 | *Escherichia coli* | 1 | ESBL | CTX-M |
| ES-16 | *Escherichia coli* | 1 | ESBL | CTX-M TEM |
| ES-17 | *Escherichia coli* | 1 | - | TEM |
| ES-18 | *Escherichia coli* | 1 | - | - |
| ES-28 | *Escherichia coli* | 1.5 | - | TEM |
| ES-29 | *Escherichia coli* | 1 | - | - |
| ES-30 | *Escherichia coli* | 1.5 | - | - |
| ES-31 | *Escherichia coli* | 1 | - | - |
| ES-32 | *Escherichia coli* | 0.78 | - | - |
| ES-34 | *Escherichia coli* | 8 | - | - |
| ES-35 | *Escherichia coli* | 2 | - | - |
| ES-36 | *Escherichia coli* | 1 | - | - |
| ES-37 | *Escherichia coli* | 1 | - | - |
| ES-38 | *Escherichia coli* | 1.5 | - | - |
| ES-39 | *Escherichia coli* | 1 | - | - |
| ES-41 | *Escherichia coli* | 2 | - | - |
| ES-42 | *Escherichia coli* | 1 | - | - |
| ES-43 | *Escherichia coli* | 1 | - | - |
| ES-44 | *Escherichia coli* | 1.5 | - | - |
| ES-45 | *Escherichia coli* | 1 | - | TEM |
| ES-46 | *Escherichia coli* | 1.5 | - | - |
| ES-47 | *Escherichia coli* | 1 | - | TEM |
| ES-48 | *Escherichia coli* | 1 | - | - |
| ES-49 | *Escherichia coli* | 1 | - | - |
| ES-50 | *Escherichia coli* | 16 | ESBL | CTX-M TEM |
| ES-51 | *Escherichia coli* | 1 | - | TEM |

| | | | | |
|---|---|---|---|---|
| ES-52 | *Escherichia coli* | 32 | ESBL | CTX-M |
| ES-53 | *Escherichia coli* | 1 | - | - |
| ES-54 | *Escherichia coli* | 1 | - | - |
| ES-55 | *Escherichia coli* | 1.5 | - | - |

**Table S2.** Individualized results of the RapidTZP test for 80 *K. pneumoniae* and *E. coli* clinical isolates obtained directly from hemocultures.

| Id. | RapidTZP | MicroScan (mg/L) | Microorganism | TZP CMI Etest | ESBL/Carbapenemase |
|---|---|---|---|---|---|
| TZP-1 | S | 8 | *Klebsiella pneumoniae* | 8 | CTX-M |
| TZP-2 | S | <8 | *Klebsiella pneumoniae* | 4 | - |
| TZP-3 | S | 3 | *Klebsiella pneumoniae* | 3 | CTX-M |
| TZP-4 | S | <8 | *Klebsiella pneumoniae* | 6 | CTX-M |
| TZP-5 | R | >16 | *Klebsiella pneumoniae* | >256 | - |
| TZP-6 | S | <8 | *Klebsiella pneumoniae* | 2 | - |
| TZP-7 | S | <8 | *Klebsiella pneumoniae* | 3 | - |
| TZP-8 | S | <8 | *Klebsiella pneumoniae* | 1,5 | - |
| TZP-9 | S | <8 | *Klebsiella pneumoniae* | 8 | CTX-M |
| TZP-10 | S | <8 | *Klebsiella pneumoniae* | 2 | - |
| TZP-11 | S | <8 | *Klebsiella pneumoniae* | 8 | CTX-M |
| TZP-12 | R | >16 | *Klebsiella pneumoniae* | 128 | - |
| TZP-13 | S | <8 | *Escherichia coli* | 2 | - |
| TZP-14 | S | <8 | *Escherichia coli* | 2 | - |
| TZP-15 | S | <8 | *Escherichia coli* | 1,5 | - |
| TZP-16 | S | <8 | *Klebsiella pneumoniae* | 3 | CTX-M |
| TZP-17 | S | <8 | *Klebsiella pneumoniae* | 8 | CTX-M |
| TZP-18 | S | <8 | *Escherichia coli* | 1,5 | - |
| TZP-19 | R | >16 | *Klebsiella pneumoniae* | >256 | - |
| TZP-20 | S | <8 | *Escherichia coli* | 4 | - |
| TZP-21 | S | <8 | *Klebsiella pneumoniae* | 2 | - |
| TZP-22 | S | <8 | *Klebsiella pneumoniae* | 8 | CTX-M |
| TZP-23 | R | 16 | *Escherichia coli* | 24 | CTX-M |
| TZP-24 | S | <8 | *Klebsiella pneumoniae* | 6 | CTX-M |
| TZP-25 | S | <8 | *Escherichia coli* | 1,5 | - |
| TZP-26 | S | <8 | *Klebsiella pneumoniae* | 2 | - |
| TZP-27 | S | <8 | *Escherichia coli* | 0,75 | - |
| TZP-28 | S | <8 | *Klebsiella pneumoniae* | 2 | CTX-M |
| TZP-29 | S | <8 | *Klebsiella pneumoniae* | 3 | CTX-M |
| TZP-30 | S | <8 | *Escherichia coli* | 2 | - |
| TZP-31 | S | <8 | *Klebsiella pneumoniae* | 8 | - |
| TZP-32 | S | <8 | *Escherichia coli* | 1 | - |
| TZP-33 | S | <8 | *Klebsiella pneumoniae* | 2 | - |
| TZP-34 | S | <8 | *Escherichia coli* | 1 | - |
| TZP-35 | S | 8 | *Klebsiella pneumoniae* | 8 | CTX-M |
| TZP-36 | S | <8 | *Escherichia coli* | 0,5 | - |
| TZP-37 | S | <8 | *Klebsiella pneumoniae* | 1,5 | - |
| TZP-38 | S | <8 | *Klebsiella pneumoniae* | 1,5 | - |
| TZP-39 | S | <8 | *Klebsiella pneumoniae* | 1,5 | - |
| TZP-40 | S | <8 | *Escherichia coli* | 1,5 | - |
| TZP-41 | S | <8 | *Escherichia coli* | 1 | - |
| TZP-42 | S | <8 | *Escherichia coli* | 3 | - |

| | | | | | |
|---|---|---|---|---|---|
| TZP-43 | S | <8 | *Escherichia coli* | 1,5 | - |
| TZP-44 | R | >16 | *Klebsiella pneumoniae* | >256 | - |
| TZP-45 | R | 16 | *Klebsiella pneumoniae* | 48 | CTX-M |
| TZP-46 | S | <8 | *Escherichia coli* | 0,75 | - |
| TZP-47 | S | <8 | *Escherichia coli* | 3 | - |
| TZP-48 | R | 48 | *Klebsiella pneumoniae* | 48 | - |
| TZP-49 | S | <8 | *Klebsiella pneumoniae* | 4 | CTX-M |
| TZP-50 | S | <8 | *Escherichia coli* | 1,5 | - |
| TZP-51 | S | <8 | *Escherichia coli* | 4 | - |
| TZP-52 | S | <8 | *Klebsiella pneumoniae* | 6 | CTX-M |
| TZP-53 | S | <8 | *Klebsiella pneumoniae* | 2 | - |
| TZP-54 | S | <8 | *Klebsiella pneumoniae* | 4 | - |
| TZP-55 | S | <8 | *Klebsiella pneumoniae* | 8 | - |
| TZP-56 | R | >16 | *Klebsiella pneumoniae* | >256 | CTX-M + OXA-48 |
| TZP-57 | S | <8 | *Escherichia coli* | 0,75 | - |
| TZP-58 | S | 1 | *Escherichia coli* | 1 | - |
| TZP-59 | S | 3 | *Klebsiella pneumoniae* | 3 | - |
| TZP-60 | S | 0.75 | *Escherichia coli* | 0,75 | - |
| TZP-61 | S | 1 | *Escherichia coli* | 1 | - |
| TZP-62 | S | 2 | *Escherichia coli* | 2 | - |
| TZP-63 | S | 1.5 | *Escherichia coli* | 1,5 | - |
| TZP-64 | S | 1 | *Escherichia coli* | 1 | - |
| TZP-65 | S | 2 | *Escherichia coli* | 2 | - |
| TZP-66 | S | <8 | *Escherichia coli* | 2 | - |
| TZP-67 | S | <8 | *Escherichia coli* | 4 | - |
| TZP-68 | S | <8 | *Escherichia coli* | 4 | - |
| TZP-69 | S | <8 | *Escherichia coli* | 2 | - |
| TZP-70 | S | <8 | *Escherichia coli* | 3 | - |
| TZP-71 | S | <8 | *Escherichia coli* | 4 | CTX-M |
| TZP-72 | S | <8 | *Klebsiella pneumoniae* | 8 | CTX-M |
| TZP-73 | S | <8 | *Escherichia coli* | 2 | - |
| TZP-74 | S | <8 | *Escherichia coli* | 0,38 | - |
| TZP-75 | S | <8 | *Klebsiella pneumoniae* | 3 | - |
| TZP-76 | S | <8 | *Klebsiella pneumoniae* | 2 | - |
| TZP-77 | S | <8 | *Escherichia coli* | 2 | - |
| TZP-78 | S | <8 | *Klebsiella pneumoniae* | 3 | CTX-M |
| TZP-79 | S | <8 | *Klebsiella pneumoniae* | 2 | - |
| TZP-80 | S | <8 | *Klebsiella pneumoniae* | 4 | CTX-M |

**Table S3**. Primers and PCR conditions for all the resistance genes tested.

| Genes | Primers | PCR type | PCR conditions |
|---|---|---|---|
| *bla*$_{CTX-M-a}$ | F:5'- CGG GCR ATG GCG CAR AC-3'<br>R:5'-TGC RCC GGT SGT ATT GCC-3' | Singleplex qPCR | 95ºC → 5 min.<br>35 x [95ºC → 15 sec.; 60ºC → 20 sec.; 72ºC → 25 sec.] |
| *bla*$_{CTX-M-b}$ | F:5'- ACC GAG CCS ACG CTC AA-3'<br>R:5'-CCG CTG CCG GTT TTA TC-3' | | |
| *bla*$_{TEM}$ | F:5'-GCA TCT TAC GGA TGG CAT GA-3'<br>R:5'-GTC CTC CGA TCG TTG TCA GAA-3' | | |
| *bla*$_{SHV}$ | F:5'-TCC CAT GAT GAG CAC CTT TAA A-3'<br>R:5'-TCC TGC TGG CGA TAG TGG AT-3' | | |
| *bla*$_{DHA}$ | F:5'-TTA ACG GTG TGA CCA ACG AG-3'<br>R:5'-ACA ATC GCC ACC TGT TTT TC-3' | | |
| *bla*$_{CMY}$ | F:5'-GGC AAA CAG TGG CAG GGT AT-3'<br>R:5'-AAT GCG GCT TTA TCC CTA ACG-3' | | |
| *bla*$_{OXA}$ | F:5'-GGA TAA AAC CCC CAA AGG AA-3'<br>R:5'-TGC ACC AGT TTT TTT CCC ATA CA-3' | PCR | 95ºC → 5 min.<br>35 x [95ºC → 30 sec.; 60ºC → 30 sec.; 72ºC → 1 min.]<br>72ºC → 5 min.<br>4ºC → ∞ |
